# Supplementary material for: Association between early-pandemic food assistance use and subsequent food security trajectories among households in Washington State during the first three years of the COVID-19 pandemic
Source: PLoS One. 2025 May 14;20(5):e0321585. doi: 10.1371/journal.pone.0321585 (PMC12077706; doi:10.1371/journal.pone.0321585)
Supplement: S1 Fig — (PDF) [file pone.0321585.s001.pdf]

S1 Figure. Analytic sample inclusion and exclusion flow diagram, WAFOOD 1-4 (2020-2023)

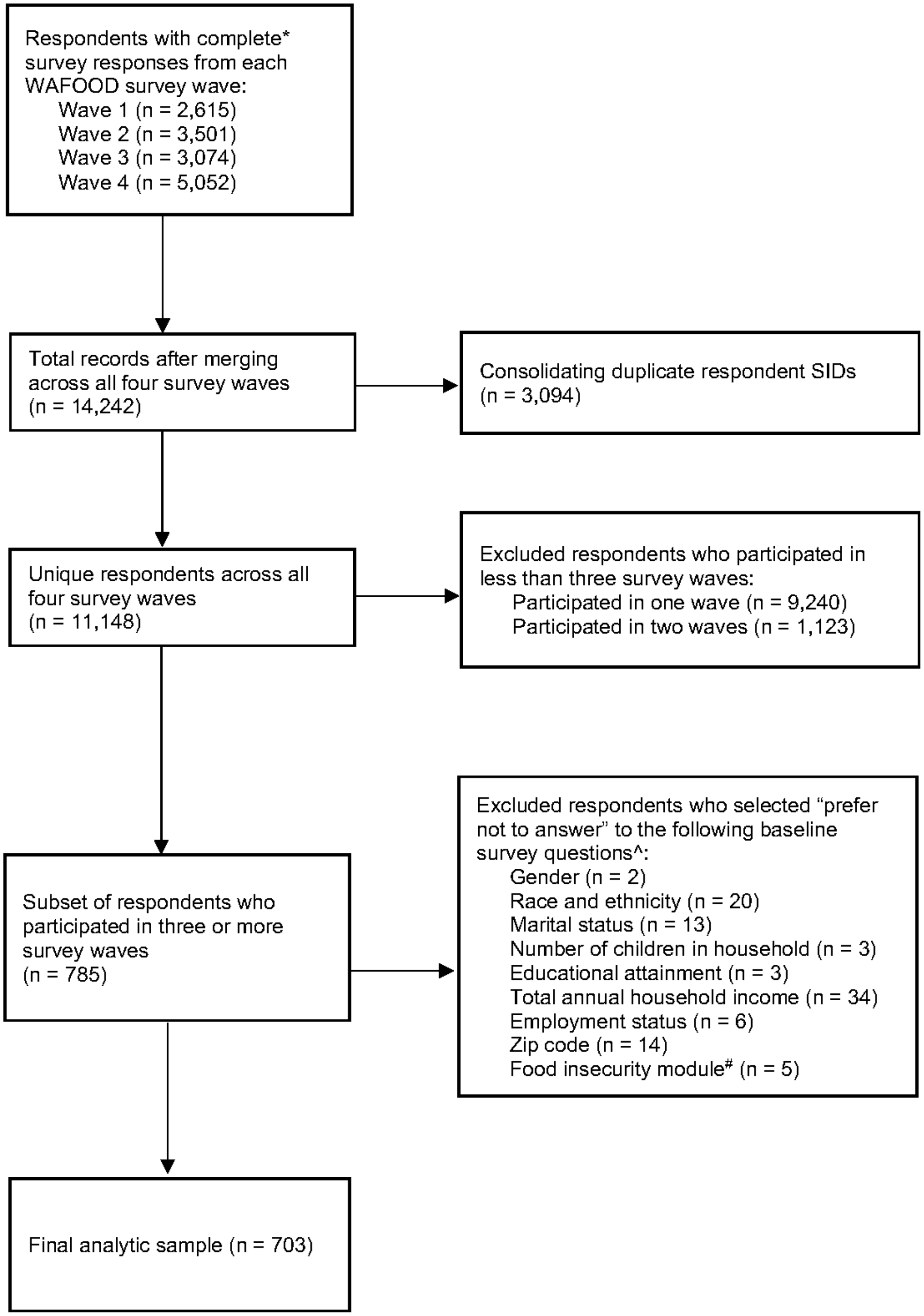

*A respondent was determined to have a complete response if they responded to all survey questions (i.e., did not terminate the survey early).

^Multiple respondents (n=12) selected "prefer not to answer" to more than one of the questions listed. Of those, five respondents selected "prefer not to answer" to three or more of the questions listed.

#Food insecurity was assessed using the validated US Household Food Security Survey Module designed by the United States Department of Agriculture Economic Research Service.
