# Supplementary material for: Association between early-pandemic food assistance use and subsequent food security trajectories among households in Washington State during the first three years of the COVID-19 pandemic
Source: PLoS One. 2025 May 14;20(5):e0321585. doi: 10.1371/journal.pone.0321585 (PMC12077706; doi:10.1371/journal.pone.0321585)

S3 Figure. Food assistance use for WAFOOD cross-sectional samples (A) and the longitudinal sample of respondents participating in three or more survey waves (B), WAFOOD 1-4 (2020-2023)

A) Food assistance use for the WAFOOD cross-sectional samples by wave

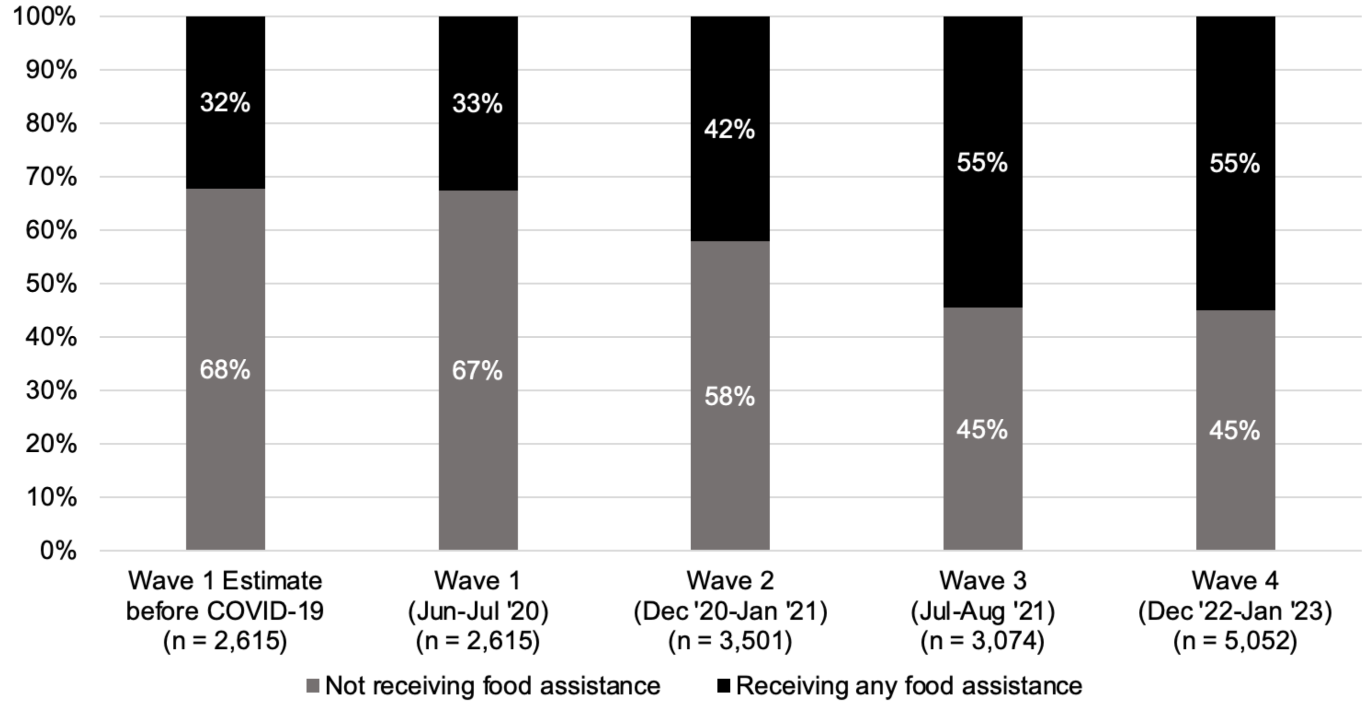

B) Food assistance use for the longitudinal sample of respondents participating in three or more survey waves

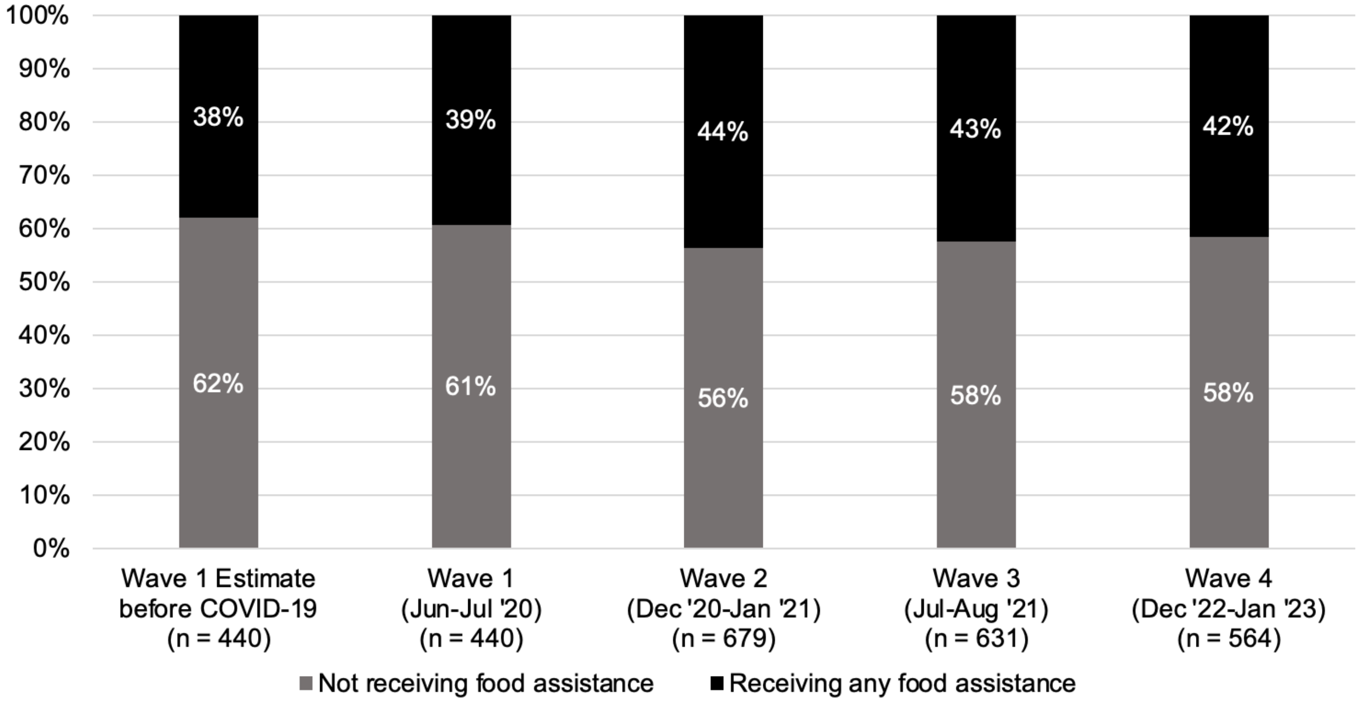

Supplement: S3 Fig — (PDF) [file pone.0321585.s003.pdf]
