# Supplementary material for: Association between early-pandemic food assistance use and subsequent food security trajectories among households in Washington State during the first three years of the COVID-19 pandemic
Source: PLoS One. 2025 May 14;20(5):e0321585. doi: 10.1371/journal.pone.0321585 (PMC12077706; doi:10.1371/journal.pone.0321585)

S4 Figure. Food security trajectories by food assistance use before COVID-19 and food assistance use at baseline and baseline WAFOOD survey wave, WAFOOD 1-4 (2020-2023)

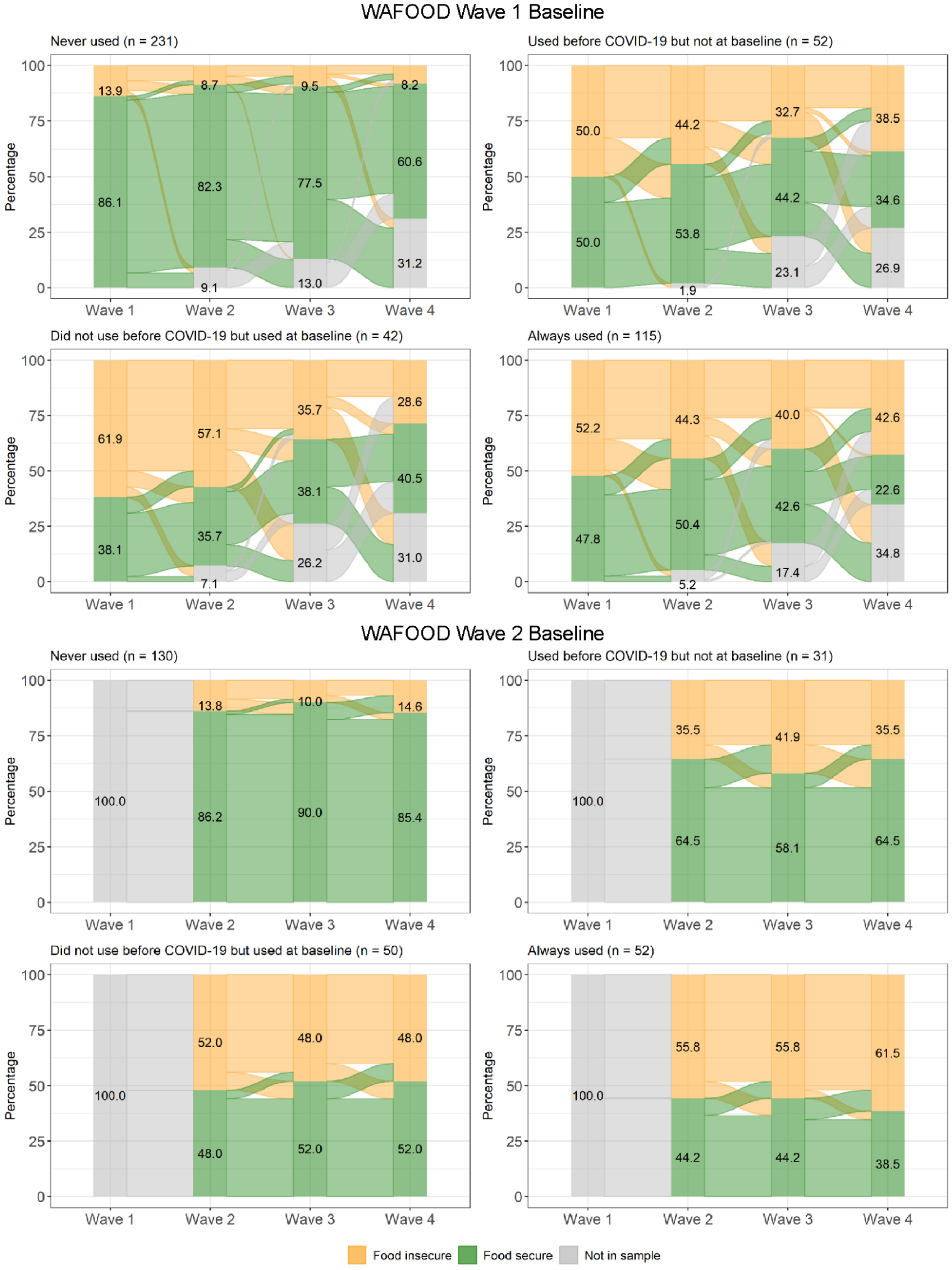

Supplement: S4 Fig — (PDF) [file pone.0321585.s004.pdf]
