# Supplementary material for: Association between early-pandemic food assistance use and subsequent food security trajectories among households in Washington State during the first three years of the COVID-19 pandemic
Source: PLoS One. 2025 May 14;20(5):e0321585. doi: 10.1371/journal.pone.0321585 (PMC12077706; doi:10.1371/journal.pone.0321585)
Supplement: S1 Table — (PDF) [file pone.0321585.s005.pdf]

S1 Table. Detailed food assistance use categories at baseline observation and food security trajectories, WAFOOD 1-4 (2020-2023) (n = 703)

	Total<br>n (%)
**Food assistance use categories**	
None	444 (63.2)
Retail only	29 (4.1)
School only	46 (6.5)
Emergency only	89 (12.7)
Retail and school	14 (2.0)
Retail and emergency	35 (5.0)
School and emergency	22 (3.1)
All three (retail, school, emergency)	24 (3.4)
**Food security trajectories**[a]	
Persistently food secure	403 (57.3)
Persistently food insecure	142 (20.2)
Experienced one or more food insecurity transitions	158 (22.5)
Transitioned to and stayed food insecure	52 (7.4)
Transitioned to and stayed food secure	52 (7.4)
Multiple transitions	54 (7.7)

[a] Estimates shown provide overall sample percentages for the food security trajectories used in the main analyses, further breaking down the category “experienced one or more food insecurity transitions.”

Note: The “retail” food assistance use category includes SNAP (Supplemental Nutrition Assistance Program, Food Stamps, Basic Food, or EBT), WIC (Women, Infants and Children), grocery vouchers or cash cards (provided by the city, food bank, food pantry, or other source), and Pandemic-EBT (for respondents whose baseline was Wave 2). The “school” food assistance use category includes school meal programs (breakfast, lunch) and summer meals from schools for children. The “emergency” food assistance use category includes food banks, mobile boxes (mobile food bank, drive-through, food gives, or other pop-up sites), food delivery from community programs, and other programs (e.g., Commodity Supplemental Food Program, Meals on Wheels).
