# Supplementary material for: Association between early-pandemic food assistance use and subsequent food security trajectories among households in Washington State during the first three years of the COVID-19 pandemic
Source: PLoS One. 2025 May 14;20(5):e0321585. doi: 10.1371/journal.pone.0321585 (PMC12077706; doi:10.1371/journal.pone.0321585)
Supplement: S2 Table — (PDF) [file pone.0321585.s006.pdf]

**S2 Table.** Associations between food assistance use before COVID-19 and at baseline and food security trajectories, WAFOOD 1-4 (2020-2023) (n = 703)

| Food assistance use before COVID-19 and at baseline | Model 1 | | Model 2 | | Model 3 | | Model 4 | |
|---|---|---|---|---|---|---|---|---|
| | PP (95% CI) | ME (95% CI) | PP (95% CI) | ME (95% CI) | PP (95% CI) | ME (95% CI) | PP (95% CI) | ME (95% CI) |
| **Persistently food insecure vs. persistently food secure (ref.) (n = 545)** | | | | | | | | |
| Never used | 0.071 (0.042, 0.099) | Ref. — | 0.081 (0.048, 0.115) | Ref. — | 0.122 (0.074, 0.170) | Ref. — | 0.121 (0.074, 0.169) | Ref. — |
| Used before COVID-19 but not at baseline | 0.407 (0.276, 0.539) | 0.337 (0.203, 0.471) | 0.346 (0.240, 0.453) | 0.265 (0.151, 0.379) | 0.236 (0.162, 0.310) | 0.114 (0.021, 0.207) | 0.239 (0.164, 0.314) | 0.118 (0.024, 0.212) |
| Did not use before COVID-19 but used at baseline | 0.515 (0.396, 0.634) | 0.444 (0.321, 0.566) | 0.474 (0.357, 0.590) | 0.392 (0.267, 0.518) | 0.444 (0.342, 0.545) | 0.322 (0.205, 0.438) | 0.441 (0.340, 0.542) | 0.320 (0.204, 0.436) |
| Always used | 0.568 (0.475, 0.660) | 0.497 (0.400, 0.594) | 0.460 (0.364, 0.557) | 0.379 (0.270, 0.489) | 0.327 (0.261, 0.393) | 0.205 (0.113, 0.297) | 0.327 (0.261, 0.393) | 0.206 (0.114, 0.298) |
| Joint *p*-value | <.001 | | <.001 | | <.001 | | <.001 | |
| **Experienced one or more food insecurity transitions vs. persistently food secure (ref.) (n = 561)** | | | | | | | | |
| Never used | 0.145 (0.107, 0.182) | Ref. — | 0.157 (0.115, 0.199) | Ref. — | 0.194 (0.140, 0.248) | Ref. — | 0.193 (0.140, 0.247) | Ref. — |
| Used before COVID-19 but not at baseline | 0.475 (0.350, 0.601) | 0.331 (0.200, 0.462) | 0.424 (0.313, 0.536) | 0.267 (0.146, 0.388) | 0.349 (0.250, 0.448) | 0.155 (0.035, 0.275) | 0.350 (0.251, 0.450) | 0.157 (0.036, 0.278) |
| Did not use before COVID-19 but used at baseline | 0.421 (0.293, 0.549) | 0.277 (0.142, 0.410) | 0.44 (0.299, 0.582) | 0.283 (0.132, 0.433) | 0.409 (0.279, 0.540) | 0.215 (0.068, 0.262) | 0.409 (0.279, 0.539) | 0.216 (0.069, 0.363) |
| Always used | 0.538 (0.443, 0.634) | 0.394 (0.291, 0.497) | 0.441 (0.347, 0.536) | 0.284 (0.174, 0.394) | 0.336 (0.258, 0.414) | 0.142 (0.034, 0.249) | 0.337 (0.258, 0.416) | 0.143 (0.034, 0.252) |
| Joint *p*-value | <.001 | | <.001 | | .007 | | .007 | |

Ref. = reference group, PP = predicted probability, CI = confidence interval, ME = marginal effect

Note: Sample sizes for each food security trajectory are "persistently food secure," n = 403; "persistently food insecure," n = 142; and "people who experienced one or more food insecurity transitions," n = 158. Sample sizes for each food assistance use category are "neither used before COVID-19 nor at baseline," n = 361; "used before COVID-19 but not at baseline," n = 83; "did not use before COVID-19 but used at baseline," n = 92; and "used both before COVID-19 and at baseline," n = 167. A respondent's "baseline" measurement was determined as either Wave 1 or Wave 2, depending on whether the first WAFOOD survey they took was at Wave 1 or Wave 2. Food assistance use included the following programs: SNAP (Supplemental Nutrition Assistance Program, Food Stamps, Basic Food, or EBT), WIC (Women, Infants and Children), grocery vouchers or cash cards (provided by the city, food bank, food pantry, or other source), Pandemic-EBT (for respondents whose baseline was wave 2), school meal programs (breakfast, lunch), summer meals from schools for children, food banks, mobile boxes (mobile food bank, drive-through, food gives, or other pop-up sites), food delivery from community programs, and other programs (e.g., Commodity Supplemental Food Program, Meals on Wheels). Model 1 is unadjusted. Model 2 adjusts for age, gender, race and ethnicity, marital status, and any children in the household at baseline. Model 3 adjusts for Model 2 and additionally adjusts for educational attainment, total annual household income, and current employment status at baseline. Model 4 adjusts for Model 3 and additionally adjusts for urbanicity at baseline.
