# Supplementary material for: Association between early-pandemic food assistance use and subsequent food security trajectories among households in Washington State during the first three years of the COVID-19 pandemic
Source: PLoS One. 2025 May 14;20(5):e0321585. doi: 10.1371/journal.pone.0321585 (PMC12077706; doi:10.1371/journal.pone.0321585)
Supplement: S3 Table — (PDF) [file pone.0321585.s007.pdf]

S3 Table. Sensitivity analyses examining the associations between food assistance use before COVID-19 and at baseline and food security trajectory outcomes, WAFOOD 1-4 (2020-2023)

Food assistance use before COVID-19 and at baseline	Restricting respondent sample to those with contiguous waves of food security data		Restricting respondent sample to those with complete food security data	
	**PP (95% CI)**	**ME (95% CI)**	**PP (95% CI)**	**ME (95% CI)**
**Persistently food insecure vs. persistently food secure (ref.)**	**(n = 465)**		**(n = 356)**	
Never used	0.120 (0.067, 0.174)	Ref. —	0.112 (0.055, 0.170)	Ref. —
Used before COVID-19 but not at baseline	0.213 (0.137, 0.290)	0.093 (-0.007, 0.194)	0.213 (0.125, 0.300)	0.100 (-0.013, 0.213)
Did not use before COVID-19 but used at baseline	0.441 (0.323, 0.557)	0.321 (0.189, 0.453)	0.424 (0.302, 0.545)	0.311 (0.172, 0.450)
Always used	0.300 (0.234, 0.367)	0.180 (0.083, 0.277)	0.318 (0.240, 0.396)	0.206 (0.096, 0.316)
Joint *p*-value	<.001		<.001	
**Experienced one or more food insecurity transitions vs. persistently food secure (ref.)**	**(n = 484)**		**(n = 371)**	
Never used	0.207 (0.146, 0.270)	Ref. —	0.212 (0.142, 0.283)	Ref. —
Used before COVID-19 but not at baseline	0.321 (0.214, 0.428)	0.113 (-0.019, 0.245)	0.335 (0.214, 0.456)	0.122 (-0.029, 0.274)
Did not use before COVID-19 but used at baseline	0.370 (0.232, 0.507)	0.162 (0.002, 0.321)	0.346 (0.202, 0.490)	0.134 (-0.040, 0.308)
Always used	0.318 (0.240, 0.396)	0.110 (-0.004, 0.225)	0.324 (0.231, 0.417)	0.111 (-0.021, 0.244)
Joint *p*-value	0.133		0.280	

Ref. = reference group, PP = predicted probability, CI = confidence interval, ME = marginal effect

Note: A respondent's "baseline" measurement was determined as either Wave 1 or Wave 2, depending on whether the first WAFOOD survey they took was at Wave 1 or Wave 2. Food assistance use included the following programs: SNAP (Supplemental Nutrition Assistance Program, Food Stamps, Basic Food, or EBT), WIC (Women, Infants and Children), grocery vouchers or cash cards (provided by the city, food bank, food pantry, or other source), Pandemic-EBT (for respondents whose baseline was wave 2), school meal programs (breakfast, lunch), summer meals from schools for children, food banks, mobile boxes (mobile food bank, drive-through, food gives, or other pop-up sites), food delivery from community programs, and other programs (e.g., Commodity Supplemental Food Program, Meals on Wheels). Estimates presented are from Model 4 and adjust for age, gender, race and ethnicity, marital status, any children in the household, educational attainment, total annual household income, current employment status, and urbanicity at baseline.
