# Supplementary material for: Association between early-pandemic food assistance use and subsequent food security trajectories among households in Washington State during the first three years of the COVID-19 pandemic
Source: PLoS One. 2025 May 14;20(5):e0321585. doi: 10.1371/journal.pone.0321585 (PMC12077706; doi:10.1371/journal.pone.0321585)
Supplement: Attachment — Submitted filename: PLOS One.docx [file pone.0321585.s010.docx]

Thank you for the opportunity to review this manuscript. I really enjoyed reading it and I think it will make an excellent contribution to the literature. The manuscript is well-written, concise, and easy to follow. I just have a few questions and comments:

Why were longitudinal regression analyses not used (e.g., generalized estimating equations)? The analyses should account for repeated measures within individuals.

Thank you for including Supplemental Figures 2 and 3 and comparing the longitudinal sample to the larger cross-sectional sample in terms of food insecurity and food assistance use. Were the two samples similar in terms of demographic characteristics? It might be helpful to readers to include one or two sentences clarifying this.

I understand the value in presenting the different models that were built, but in the Results section, there’s a lot of focus on the unadjusted models and I’m not sure why. It seems like there should be more of a focus (or a complete focus) on the adjusted models, particularly the fully adjusted model. Can the authors clarify why they present and interpret results from the unadjusted models when those coefficients could be biased due to confounding?

How many questions was the WAFOOD, and which WAFOOD questions came from validated preexisting surveys (e.g., USDA Household Food Security Survey Module, BRFSS, NHANES) and which ones were new? How long did it take participants to complete the survey?

I appreciate the authors acknowledging the convenience sample as a limitation throughout the manuscript. It might be useful to readers to expand on this more in the Discussion- for example, if this sample *was* representative (i.e., *not* mostly urban, college educated white women), how might the results change? Would the associations be even stronger (especially given that the sample may have missed households without internet access or computers, cell phones, or tablets, which could be a proxy for low-income/high food insecurity)? It might also be helpful to specifically state in the limitations that this sample was mostly urban white women (e.g., modifying this sentence to something like: First, WAFOOD respondents were not representative of all WA residents; they were mostly urban, white women, while the greater WA state population consists of a much larger population of _____.”).

I think the Discussion section would benefit from a deeper discussion on *why* certain demographic characteristics (e.g., age [35 to 64 years of age], race/ethnicity [non-Hispanic Black], marital status [being single or divorced], gender identity) may have had the highest proportion of food insecurity or experiencing a food insecurity transition, and what are the policy implications of these results. For example, maybe higher age was protective because there were special programs for seniors that protected against food insecurity during the pandemic, such as Meals on Wheels, free grocery delivery for seniors, or just the fact that many seniors live in assisted living homes or live with caretakers who can cook and shop for them (e.g., other family members). Perhaps food assistance programs should therefore focus more on assisting younger populations. You could also cite that other studies have found that higher age may have been protective against food insecurity during the pandemic (e.g., [Chapman et al.](https://www.tandfonline.com/doi/full/10.1080/19320248.2023.2202618) found that starting at age 60, higher age became protective against food insufficiency during the first year of the COVID-19 pandemic, such that participants ages 60–69, 70–79, and 80+ had a 13%, 49%, and 48% lower odds of food insufficiency, respectively.).

“National unemployment jumped to 14.7% in April 2020” - jumped from what to 14.7%? Was this a large jump? I’m assuming so, but it would be helpful to clarify.

“It is estimated that during 2020, at least 60 million Americans used food banks, food pantries, or other private food assistance programs…” compared to how many normally? Is 60 million a large jump? Again, I assume so, but might be helpful to clarify.

Figure 1 is blurry- can the authors improve the quality so it is more readable?

“some impacts, like food price inflation, have lingered.” I think this may need a citation- perhaps: <https://www.ers.usda.gov/data-products/ag-and-food-statistics-charting-the-essentials/food-prices-and-spending/?topicId=1afac93a-444e-4e05-99f3-53217721a8be> (“US food prices rose by 25 percent from 2019 to 2023”).

“This study uses data from residents across Washington State (WA), which was

the site of the first US COVID-19 death in March 2020.” – why is this important? Are the authors implying that WA State may have been hit more intensely than other states since they were the site of the first death and therefore dealt with the consequences of COVID-19 for longer?

“Each respondent received a raw score based on their answers, which we used to assign food security categories. Scores of 0 or 1 indicated high or marginal food security, scores of 2-4 indicated low food security, and scores of 5 or 6 indicated very low food security. We dichotomized scores into food secure if respondents had high or marginal food security and food insecure if respondents had low or very low food security.” – I think you can state that this is in accordance with the USDA ERS’s standard scoring practices and then cite this (page 4, where it says “For some reporting purposes, the food security status of households with raw score 0-1 is

described as food secure and the two categories “low food security” and “very low food security” in combination are referred to as food insecure.”): <https://www.ers.usda.gov/media/8282/short2012.pdf>. I think this might be your Reference #28. Just a suggestion so that readers don’t think you’re changing the survey’s scoring practices.
