# Supplementary material for: Association between early-pandemic food assistance use and subsequent food security trajectories among households in Washington State during the first three years of the COVID-19 pandemic
Source: PLoS One. 2025 May 14;20(5):e0321585. doi: 10.1371/journal.pone.0321585 (PMC12077706; doi:10.1371/journal.pone.0321585)
Supplement: Attachment — Submitted filename: FI_trj_PLOS_ONE_R&R_20250204_v3.docx [file pone.0321585.s012.docx]

**Department of Epidemiology** ▪ James Buszkiewicz, Ph.D., MPH ▪ Research Assistant Professor

Karen M Davison, Ph.D.

Academic Editor

*PLOS ONE*

4 February 2025

Dr. Davison and reviewers,

Thank you for your consideration of our manuscript, “Associations between early-pandemic food assistance use and subsequent food security trajectories among households in Washington State during the first three years of the COVID-19 pandemic,” for publication in *PLOS ONE* and for the opportunity to revise our manuscript. We have made every effort to address the reviewers’ feedback point by point as thoroughly as possible. Specially, we have re-run our primary analysis using a modified Poisson regression, calculating and visualizing predicted probabilities and marginal effects of the association between food assistance use patterns before COVID-19 and at baseline and subsequent food insecurity trajectories, adjusting for sociodemographic factors. In addition, we have added sensitivity analyses testing the robustness of our findings to additional restrictions based on missing food security information and comparing our analytic sample to the larger, cross-sectional Washington Food Security Survey waves and to Washington State residents and households. To more thoroughly respond to reviewers, we also added an additional visualization of the food insecurity trajectories observed in our respondent sample using alluvial plots generated using the R statistical package and revised Table 3 to more accurately reflect the food assistance use pattern categories used in our main associational analysis. All of these changes have been highlighted in yellow in the ‘tracked’ version of the manuscript. Finally, we made minor edits throughout to further improve the clarity and conciseness of our manuscript.

We believe that the reviewers’ feedback has greatly strengthened our manuscript and we believe it will be of great value to the readers of *PLOS ONE*. We look forward to your response and decision.

Sincerely,

Reviewer #1

Thank you for the opportunity to review this manuscript. I really enjoyed reading it and I think it will make an excellent contribution to the literature. The manuscript is well-written, concise, and easy to follow. I just have a few questions and comments:

We thank Reviewer #1 for their kind words and for their time in providing a thorough review and thoughtful feedback on our manuscript.

Why were longitudinal regression analyses not used (e.g., generalized estimating equations)? The analyses should account for repeated measures within individuals.

The reviewer makes an excellent point, and we completely agree. Our original analysis, conducted in R, did not allow us to account for repeated measures using ‘mlogit.’ We now conduct the analyses using Stata 18. We have further opted to use generalized estimating equations using the family Poisson, log link, and an independent correlation matrix to account for repeated observations nested within respondents, along with robust standard errors (a modified Poisson regression model). We also use the Stata ‘margins’ post-estimation command to calculate the predicted probabilities of *persistently food insecure* or experiencing *one or more food insecurity transitions* relative to being *persistently food secure* over each food assistance use pattern. We also calculate the difference in these predicted probabilities or the ‘marginal effect’ for each food assistance use pattern relative to having never used food assistance (before COVID-19 or at baseline).

We describe this new analysis on page 9, paragraph 1 of the ‘Statistical analysis’ sub-section of the ‘Methods’ section. We describe the results of these new analyses on page 16, paragraphs 2 and 3 of the ‘Association between early-pandemic food assistance use and food security trajectories’ sub-section of the ‘Results’ section. Full main association estimates are presented in Supplemental Table 2 while a visual representation of results from the fully adjusted Model 4 are presented in Figures 2 and 3.

Thank you for including Supplemental Figures 2 and 3 and comparing the longitudinal sample to the larger cross-sectional sample in terms of food insecurity and food assistance use. Were the two samples similar in terms of demographic characteristics? It might be helpful to readers to include one or two sentences clarifying this.

We agree with the reviewer that including a table comparing the sociodemographic characteristics of our sample to the larger WAFOOD Wave 1 and 2 cross-sectional samples would be useful context for readers in addition to the information we have already provided regarding food insecurity and food assistance use. In Supplemental Table 4 we now include descriptive statistics of our longitudinal sample, based on WAFOOD wave of entry (1 or 2) and the WAFOOD Wave 1 and 2 cross-sectional samples as well as to Washington State residents and households in 2020 using US Census bureau decennial census data and US Census American Community Survey 5-year estimates. A comparison to the aggregate longitudinal analytic sample can be achieved by comparing these descriptive statistics to Table 1.

We provide a description of this new analysis in paragraph 1 on page 11 of the ‘Sensitivity analysis’ sub-section of the ‘Methods’ section:

“Finally, we qualitatively compared the baseline sociodemographic characteristics of respondents included in our analytic sample to those respondents included in the larger WAFOOD Wave 1 and 2 cross-sectional samples and to WA residents and households using 2020 decennial U.S. Census Bureau^39^ data and U.S. Census Bureau American Community Survey 5-year estimates.^40^.”

We describe the results on paragraph 1 on page 18 and 19 of the ‘Sensitivity analysis’ sub-section of the ‘Results’ section:

“Estimates derived from sensitivity analyses restricting our sample to those respondents 1) with contiguous waves of food security data and 2) with complete food security data across waves were similar in direction and magnitude compared with primary findings (Supplemental Table 3). Compared to respondents included in the larger cross-sectional WAFOOD Wave 1 and 2 samples, a higher proportion of respondents in the longitudinal analytic sample presented here were 35 or older, non-Hispanic White, or had no children (Supplemental Table 4). A larger proportion of respondents in the longitudinal sample who entered in WAFOOD Wave 1 also had a college degree or more. However, it is important to note that sociodemographic patterns across samples were identical and the cross-sectional survey samples have some item-specific non-response. Finally, compared to WA residents in 2020,^39^ the longitudinal analytic sample presented here had a larger proportion of older, female, and non-Hispanic White adults and adults with low educational attainment and incomes below $75,000. Our sample also had more households with children than WA households in 2020.”

I understand the value in presenting the different models that were built, but in the Results section, there’s a lot of focus on the unadjusted models and I’m not sure why. It seems like there should be more of a focus (or a complete focus) on the adjusted models, particularly the fully adjusted model. Can the authors clarify why they present and interpret results from the unadjusted models when those coefficients could be biased due to confounding?

We again completely agree with the reviewer. We have completely reorganized the results section to now focus solely on associations estimated from fully adjusted models. We describe the results of these new analyses on page 17, paragraphs 1 and 2 of the ‘Associations between early-pandemic food assistance use and food security trajectories’ sub-section of the ‘Results’ section and present them visually in Figures 2 and 3. We think the focus on fully adjusted Model 4 results and presenting the findings in figures makes the results and narrative clearer. Estimates for Models 1-3 along with full Model 4 estimates are preserved and presented in Supplemental Table 2.

In addition, to test the robustness of our findings, we now also include two additional sensitivity analyses that examine the impact of missing food security data on these estimates by further restricting our analytic sample to 1) those respondents with contiguous waves of food security data and 2) respondents with complete food security data across waves. We provide a description of this new analysis in paragraph 1 on page 10 and 11 of the ‘Sensitivity analysis’ sub-section of the ‘Methods’ section:

“We tested the robustness of our findings regarding the inclusion of respondents with missing food insecurity data. First, we restricted our sample to only those respondents with contiguous waves of food security data (i.e., no gaps between waves) (n = 465). Second, we restricted our analysis to only those respondents with complete food security data (i.e., respondents were excluded who chose “prefer not to answer” across food security questions) across waves (n = 356).”

We describe the results on paragraph 1 on page 18 of the ‘Sensitivity analysis’ sub-section of the ‘Results’ section:

“Estimates derived from sensitivity analyses restricting our sample to those respondents 1) with contiguous waves of food security data and 2) with complete food security data across waves were similar in direction and magnitude compared with primary findings (Supplemental Table 3).”

How many questions was the WAFOOD, and which WAFOOD questions came from validated preexisting surveys (e.g., USDA Household Food Security Survey Module, BRFSS, NHANES) and which ones were new? How long did it take participants to complete the survey?

59 of the 72 questions of the WAFOOD survey were taken from existing surveys, with the remaining questions generated anew by this study team. The survey was designed to take approximately 20 minutes.

We now include this information in paragraph 1 and 2 on page 5 in the ‘Study design and population’ sub-section of the ‘Methods’ section:

“Of the 72 questions included in the survey, 59 (82%) were adapted from existing tools that came from the US Department of Agriculture’s (USDA) Economic Research Service (ERS) Household Food Security Survey Module,^29,30^ the Behavioral Risk Factor Surveillance System,^31^ the National Health and Nutrition Examination Survey,^32^ and surveys developed by our partners in the Nutrition and Obesity Policy Research and Evaluation Network,^33^ the National Food Access and COVID Research Team,^34^ and the Center for Nutrition and Health Impact,^35^ with the remaining 13 questions created by the study team.”

“Respondents could access the WAFOOD survey, implemented online using REDCap,^34^ using their computer, cellphone, or tablet. The survey was designed to take approximately 20 minutes.”

I appreciate the authors acknowledging the convenience sample as a limitation throughout the manuscript. It might be useful to readers to expand on this more in the Discussion- for example, if this sample *was* representative (i.e., *not* mostly urban, college educated white women), how might the results change? Would the associations be even stronger (especially given that the sample may have missed households without internet access or computers, cell phones, or tablets, which could be a proxy for low-income/high food insecurity)? It might also be helpful to specifically state in the limitations that this sample was mostly urban white women (e.g., modifying this sentence to something like: First, WAFOOD respondents were not representative of all WA residents; they were mostly urban, white women, while the greater WA state population consists of a much larger population of _____.”).

We thank the reviewer for the suggestion. As mentioned above, in Supplemental Table 4, we now provide a direct comparison to 2020 Washington State residents and households as a whole. This comparison illustrates that compared to Washington State residents and households in 2020, our population had higher representation from older, female, non-Hispanic White, less educated adults, as well as higher representation from households with children and incomes below $75,000. While the reviewer’s suggestion that excluding households without internet access or electronic devices would like lead to an underestimation of ‘true’ population-level associations, we believe that given our respondent sample’s sociodemographic profile, estimates would likely be attenuated if the sample had been state representative. However, we do not wish to speculate on what we would have found had our sample been representative, rather we would like to focus on the strengths of our sample recruited in partnership with local and state hunger relief organizations.

We include the following text in paragraph 1 on page 19 of the ‘Sensitivity analysis’ sub-section of the ‘Results’ section:

“Finally, compared to WA residents in 2020,^39^ the longitudinal analytic sample presented here had a larger proportion of older, female, and non-Hispanic White adults and adults with low educational attainment and incomes below $75,000. Our sample also had more households with children than WA households in 2020.”

We also include the following text in paragraph 5 on page 23 of the ‘Discussion’ section:

“However, it was this sampling strategy that enabled us to recruit households most burdened by food insecurity and map and describe food security trajectories”

I think the Discussion section would benefit from a deeper discussion on *why* certain demographic characteristics (e.g., age [35 to 64 years of age], race/ethnicity [non-Hispanic Black], marital status [being single or divorced], gender identity) may have had the highest proportion of food insecurity or experiencing a food insecurity transition, and what are the policy implications of these results. For example, maybe higher age was protective because there were special programs for seniors that protected against food insecurity during the pandemic, such as Meals on Wheels, free grocery delivery for seniors, or just the fact that many seniors live in assisted living homes or live with caretakers who can cook and shop for them (e.g., other family members). Perhaps food assistance programs should therefore focus more on assisting younger populations. You could also cite that other studies have found that higher age may have been protective against food insecurity during the pandemic (e.g., [Chapman et al.](https://www.tandfonline.com/doi/full/10.1080/19320248.2023.2202618) found that starting at age 60, higher age became protective against food insufficiency during the first year of the COVID-19 pandemic, such that participants ages 60–69, 70–79, and 80+ had a 13%, 49%, and 48% lower odds of food insufficiency, respectively.).

We completely agree with the reviewer and have added a new paragraph in the discussion section which attempts to highlight some of the reasons why certain sociodemographic groups may be more heavily impacted by persistent or transitory food insecurity and what the policy implications of these findings are. For the sake of brevity, we have chosen to only give a couple of examples rather than provide rationale and policy implications for all subpopulations examined in this manuscript. We have chosen to highlight racial and ethnic differences and age differences, thanks to the reviewer’s suggestion.

In paragraph 3 on page 20 and 21 in the Discussion section we have added the following text:

“That we found both persistent and transitional food insecurity to be more common in some sociodemographic groups but not others is reflective of safety net system strengths and gaps alongside the complex interplay of social, economic, and structural forces driving U.S. food insecurity.^45,46^ In line with prior work,^47,48^ households with children in our sample had a higher probability of being persistently food insecure or experiencing one or more food insecurity transitions. Before COVID-19, targeted policy efforts reduced child food insecurity to 13.6% in 2019, but economic shocks in 2020 caused a spike that was briefly alleviated to 12.5% by government waivers in existing safety net programs (e.g., expanding WIC options, maximizing SNAP allotments) and pandemic-era programs (e.g., Pandemic EBT, Child Tax Credit); however, the expiration of some waivers and of pandemic-era programs led to a sharp increase to 17.3% in 2022.^45,46^ Likewise we observed that food insecurity transitions and persistent food insecurity were more common among non-Hispanic Black and Hispanic respondents and respondents of low socioeconomic status.^45,46,49,50^ During COVID-19, these populations experienced higher unemployment ^49–51^ and higher case rates and deaths^51^ related to COVID-19^52^ compared to their non-Hispanic White and high socioeconomic counterparts, continuing pre-pandemic trends. Conversely, adults over the age of 65 in this analysis experienced fewer food insecurity transitions and were less likely to be persistently food insecure, a finding that has been reflected in other work, which may be due to the higher availability of food assistance programs for older rather than younger adults.^53^ Our work, in the context of a rich body of work examining U.S. food insecurity ^54,55^ highlights opportunities to build a more resilient food system and hunger relief program network through partnerships between researchers, hunger relief organizations, government organizations, and policymakers to identify populations in need of targeted policies and interventions.”

References

Bowen S, Elliott S, Hardison-Moody A. The structural roots of food insecurity: How racism is a fundamental cause of food insecurity. Sociol Compass. 2021;15(7):e12846. doi:https://doi.org/10.1111/soc4.12846

Tan S Bin, deSouza P, Raifman M. Structural Racism and COVID-19 in the USA: a County-Level Empirical Analysis. J Racial Ethn Health Disparities. 2022;9(1):236-246. doi:10.1007/s40615-020-00948-8

Chapman LE, Hu J, Seidel S. COVID-19 Cases are Associated with Food Insufficiency in the United States During the COVID-19 Pandemic. J Hunger Environ Nutr. 2023;18(3):327-342. doi:10.1080/19320248.2023.2202618

Norris K, Jilcott Pitts S, Reis H, Haynes-Maslow L. A Systematic Literature Review of Nutrition Interventions Implemented to Address Food Insecurity as a Social Determinant of Health. Nutrients. 2023;15(15). doi:10.3390/nu15153464

Alaimo K. Food Insecurity in the United States: An Overview. Top Clin Nutr. 2005;20(4). https://journals.lww.com/topicsinclinicalnutrition/fulltext/2005/10000/food_insecurity_in_the_united_states__an_overview.2.aspx

Dennard E, Kristjansson E, Tchangalova N, Totton S, Winham D, O’Connor A. Food insecurity among African Americans in the United States: A scoping review. *PLoS One*. 2022;17(9):e0274434-. https://doi.org/10.1371/journal.pone.0274434

Nchako C. Food Insecurity Rises for the Second Year in a Row. Center on Budget and Policy Priorities, Off the Charts. September 6, 2024. Accessed February 2, 2025. https://www.cbpp.org/blog/food-insecurity-rises-for-the-second-year-in-a-row

Hall L. Food Insecurity Increased in 2022, With Severe Impact on Households With Children and Ongoing Racial Inequities. Center on Budget and Policy Priorities, Off the Charts. October 26, 2023. Accessed February 2, 2025. https://www.cbpp.org/blog/food-insecurity-increased-in-2022-with-severe-impact-on-households-with-children-and-ongoing

Houghtaling B, Haynes-Maslow L, Andress L, et al. Food insecurity among households with children during the early months of the COVID-19 pandemic. *J Agric Food Syst Community Dev*. 2023;12(3):225–237. doi:10.5304/jafscd.2023.123.015

Williams A, Alwan NA, Taylor E, Smith D, Ziauddeen N. The COVID-19 pandemic and food insecurity in households with children: A systematic review. *PLoS One*. 2024;19(8):e0308699-. https://doi.org/10.1371/journal.pone.0308699

Geranios K, Kagabo R, Kim J. Impact of COVID-19 and Socioeconomic Status on Delayed Care and Unemployment. *Health Equity*. 2022;6(1):91-97. doi:10.1089/heq.2021.0115

“National unemployment jumped to 14.7% in April 2020” - jumped from what to 14.7%? Was this a large jump? I’m assuming so, but it would be helpful to clarify.

We have modified the text in paragraph 1 on page 2 in the ‘Introduction’ section to now read:

“National unemployment jumped to 13.0% in the second quarter of 2020 compared to 3.8% in the first quarter of 2020,^5^ and employment disruptions took many forms: some faced abrupt job loss during initial business closures, while others faced prolonged reduced job hours.^1,4,6^”

Please note that we now use a different citation source from the Bureau of Labor Statistics which provides different, but nevertheless, illustrative unemployment estimates:

Bureau of Labor Statistics. Unemployment rises in 2020, as the country battles the COVID-19 pandemic. Monthly Labor Review. June 2021. Accessed January 23, 2025. https://www.bls.gov/opub/mlr/2021/article/unemployment-rises-in-2020-as-the-country-battles-the-covid-19-pandemic.htm

“It is estimated that during 2020, at least 60 million Americans used food banks, food pantries, or other private food assistance programs…” compared to how many normally? Is 60 million a large jump? Again, I assume so, but might be helpful to clarify.

We have now modified the text on in paragraph 2 on page 2 in the ‘Introduction’ section to read:

“It is estimated that during 2020, at least 60 million Americans used food banks, food pantries, or other private food assistance programs, an increase of over 50% compared to 2019.^7^ Also, in 2020, the charitable food system distributed 44% more meals than the prior year.^7^”

Figure 1 is blurry- can the authors improve the quality so it is more readable?

We apologize that Figure 1 appears so blurry as we made every effort to upload a high-quality TIFF file, we noticed this as well. However, this appears to be by design from the journal. In the PDF of the compiled manuscript file, if you click the link labeled “Click here to access/download;Figure;Figure.tiff” you can download the high-quality image file. We hope that that is the way the file will appear in the final publication but will be sure to follow-up with the journal to make sure this is the case.

“some impacts, like food price inflation, have lingered.” I think this may need a citation- perhaps: <https://www.ers.usda.gov/data-products/ag-and-food-statistics-charting-the-essentials/food-prices-and-spending/?topicId=1afac93a-444e-4e05-99f3-53217721a8be> (“US food prices rose by 25 percent from 2019 to 2023”).

We thank the reviewer for the suggestion and citation and have added to paragraph 4 on page 3 in the ‘Introduction’ section.

“This study uses data from residents across Washington State (WA), which was

the site of the first US COVID-19 death in March 2020.” – why is this important? Are the authors implying that WA State may have been hit more intensely than other states since they were the site of the first death and therefore dealt with the consequences of COVID-19 for longer?

No, we added this text not to suggest that WA was hit more intensely than other states since it was the site of the first death nor the consequences of COVID-19 were more strongly felt than in other states. The COVID-19 pandemic was a traumatic national event that ebbed and flowed across, and continues to impact, states over time. We simply wished to provide additional context and motivation for the study both in its importance and in the real-time development on this initially rapid-response survey by the study’s research team, as well as the state policy environment which was very proactive in attempting to mitigate impacts of the pandemic on Washington residents. However, we believe that Figure 1 does a much better job at providing this context and a much more detailed timeline of events motivating the importance of this study. Therefore, for conciseness we removed this text.

“Each respondent received a raw score based on their answers, which we used to assign food security categories. Scores of 0 or 1 indicated high or marginal food security, scores of 2-4 indicated low food security, and scores of 5 or 6 indicated very low food security. We dichotomized scores into food secure if respondents had high or marginal food security and food insecure if respondents had low or very low food security.” – I think you can state that this is in accordance with the USDA ERS’s standard scoring practices and then cite this (page 4, where it says “For some reporting purposes, the food security status of households with raw score 0-1 is described as food secure and the two categories “low food security” and “very low food security” in combination are referred to as food insecure.”): <https://www.ers.usda.gov/media/8282/short2012.pdf>. I think this might be your Reference #28. Just a suggestion so that readers don’t think you’re changing the survey’s scoring practices.

The reviewer makes a great point, we have now added the suggested text in paragraph 1 on page 7 in the ‘Food insecurity and food security trajectories’ sub-section of the ‘Methods’ section:

“In accordance with the USDA ERS’s standard scoring practices,^27,28^ each respondent received a raw score based on their answers, which we used to assign food security categories.”

Reviewer #2: This is a wonderfully put-together research article that highlights a significant public health issue. The results were displayed in an easy-to-read, approachable manner.
No changes seem to be required.

We thank Reviewer #2 for their kind words and for their time in providing a thorough review of our work.
